# Immune markers mediate genetic relationships between immune diseases and psychiatric disorders

**DOI:** 10.1371/journal.pgen.1012328

**Published:** 2026-09-30

**Authors:** Sophie Breunig, Kristen M. Kelly, Lukas S. Schaffer, Jeremy M. Lawrence, Younga Heather Lee, Elizabeth W. Karlson, Andrew D. Grotzinger

**Affiliations:** 1 Institute for Behavioral Genetics, University of Colorado Boulder, Boulder, Colorado, United States of America; 2 Department of Psychology and Neuroscience, University of Colorado Boulder, Boulder, Colorado, United States of America; 3 Psychiatric and Neurodevelopmental Genetics Unit, Center for Genomic Medicine, Massachusetts General Hospital, Boston, Massachusetts, United States of America; 4 Department of Psychiatry, Harvard Medical School, Boston, Massachusetts, United States of America; 5 Stanley Center for Psychiatric Research, Broad Institute of MIT and Harvard, Cambridge, Massachusetts, United States of America; 6 Massachusetts General Hospital Brigham, Boston, Massachusetts, United States of America; 7 Department of Medicine, Brigham and Women’s Hospital, Harvard Medical School, Boston, Massachusetts, United States of America; 8 Department of Psychology, Memorial University, St. John’s, Newfoundland and Labrador, Canada; Moffitt Cancer Center, UNITED STATES OF AMERICA

## Abstract

This study explored the genetic links between 11 immune-mediated diseases and 13 psychiatric disorders, addressing the limited understanding of the underlying biological mechanisms linking these illnesses. Utilizing genomic structural equation modeling, we investigated whether the genetic liability for 14 immune markers (e.g., C-reactive protein [CRP], interleukin-6 [IL-6]) mediated associations between latent genomic factors of immune-mediated diseases (e.g., autoimmune) and psychiatric disorders (e.g., internalizing). While the literature extensively describes the psychiatric relevance of CRP, results indicated this is better explained by its biological precursor, IL-6. This is evidenced by highly attenuated associations of psychiatric traits with CRP when accounting for overlapping signal of IL6. Overall, the genetic liability of six immune markers significantly mediated 11 psychiatric-immune disease genetic relationships. For example, IL-6 mediated 60% of the genetic link between an internalizing factor and Crohn’s disease. Identified markers often evinced mediating effects for multiple disorder clusters and many immune-psychiatric relationships were mediated by multiple markers. At the same time, highlighting their diagnostic relevance, only a subset of examined immune markers were significant mediators. These findings provide mechanistic insight by pinpointing the immune markers that may be most critical to the psychiatric-immune disease link, which notably did not include CRP.

## Author summary

In this study, we investigated the long-observed link between immune-mediated diseases, such as Crohn’s disease or lupus, and psychiatric disorders like depression and anxiety. While the connection between the immune system and mental health is well-documented, the specific biological pathways that connect these two areas remain unclear. To address this, we used large-scale genetic data to examine whether the genetic liability for specific immune markers—proteins that signal activity in the immune system—act as mediators in these relationships. Our findings revealed that only a specific subset of immune markers, most notably Interleukin-6 (IL-6), significantly explained the genetic overlap between these conditions. Interestingly, while C-reactive protein (CRP) is frequently cited in medical literature as a key biomarker for psychiatric risk, our analysis showed that its influence is likely a byproduct of its relationship with IL-6. When we accounted for IL-6, the independent effect of CRP largely disappeared. These results help refine our understanding of the biological mechanisms driving the co-occurrence of immune and psychiatric illnesses. By identifying the most relevant markers, we hope to guide future research toward more precise diagnostic tools and targeted treatments.

## Introduction

Autoimmune and inflammatory diseases, collectively referred to as immune-mediated diseases, are conditions in which an abnormal immune response leads to prolonged activation of immune and inflammatory pathways. A growing body of literature describes high levels of comorbidity across a wide range of immune-mediated diseases and psychiatric disorders [1–3]. In line with the pervasive diagnostic overlap observed at the phenotypic level, recent genomic findings revealed that genetic overlap across an individual psychiatric disorder and immune-mediated disease often indexed broader pathways of risk sharing [4]. For example, while schizophrenia and bipolar disorder have each been separately linked to ulcerative colitis and Crohn’s disease [5–7], these risk pathways have now been shown to be shared across all four disorders [4]. The current study aims to build on this prior work to broaden our understanding of possible intermediate links between immune-mediated diseases and psychiatric disorders.

Immune and inflammatory markers are plausible mediating mechanisms given prior findings linking these markers to both immune diseases and psychiatric disorders. With respect to immune-mediated disease, these disease states are characterized by chronic activation of the immune system that is reflected in the relative increase or decrease of certain markers of immune and inflammatory activity [8–10]. An extensive prior literature has described links between these biomarkers and several immune-mediated diseases. For example, tumor necrosis factor alpha (TNF-α) has been found to be positively genetically correlated with a host of different immune-mediated diseases [11]. Immune and inflammatory markers have also been widely associated with psychiatric disorders. This includes relationships described between interleukin-1 (IL-1), interleukin-6 (IL-6), and C-reactive protein (CRP) with increased levels in depression in a dose-response fashion, where greater levels of the markers were associated with greater severity of depression symptoms [12]. Altered levels of immune markers have been reported for a wide range of psychiatric disorders, including generalized anxiety disorder [13], schizophrenia [14], bipolar disorder [15], and attention-deficit/hyperactivity disorder (ADHD) [16].

Convergent lines of evidence linking psychiatric disorders, immune-mediated diseases, and immune markers indicate that critical knowledge can be gained by jointly examining these three sets of outcomes. A limiting factor for this specific line of questioning reflects pragmatic difficulties in collecting a participant sample that has enough observations for even a single psychiatric disorder, immune-mediated disease, and immune marker. Here, we utilize the unique abilities of genetic data to bring together non-overlapping datasets by examining genetic overlap at the level of the effects of genetic variants, thereby circumventing these practical difficulties. We specifically apply a multivariate genomic approach, genomic structural equation modeling (genomic SEM), and its extensions to evaluate whether the genetic liability for a comprehensive set of immune markers mediates genetic relationships between a host of immune and psychiatric disorders.

## Results

### Psychiatric and immune factor structure

The current analyses utilized genome-wide association study (GWAS) summary statistics of 11 immune-mediated diseases, 13 psychiatric disorders, and 14 immune markers (Table 1). We began by using Genomic SEM to model the factor structure of the psychiatric disorders and immune diseases based on genetic correlations derived from linkage disequilibrium score regression (LDSC). The psychiatric factor structure reflected a previously established five-factor model [42], now including updated GWAS data for bipolar disorder, major depression, and post-traumatic stress disorder (PTSD) (Method). We found that this five-factor model continued to fit the data well with the updated data (CFI = .97; SRMR = .10). The psychiatric factors could be approximately described as reflecting compulsive, schizophrenia/bipolar, neurodevelopmental, internalizing, and substance use disorders. The immune-mediated disease factor structure consisted of a previously established four-factor model [4] (CFI = .96; SRMR = .08), with the four factors reflecting autoimmune, celiac, mixed immune pattern (denoting a shared involvement of the adaptive and innate immune system), and autoinflammatory diseases (Fig 1).

**Table 1:** Sample characteristics of included traits.

Phenotype	Cases	Controls	h^2^
***Immune Markers***			
C-C motif chemokine 2 (CCL2) [17]	46 644	–	8%
C-C motif chemokine 3 (CCL3) [17]	46 643	–	15%
C-reactive protein (CRP) [18]	427 367	–	15%
Interferon gamma (IFN-γ) [17]	46 643	–	5%
Interleukin 10 (IL-10) [17]	46 643	–	5%
Interleukin 1 beta (IL-1β) [17]	46 643	–	5%
Interleukin 1 receptor antagonist (IL-1RA) [17]	46 643	–	15%
Interleukin 6 (IL-6) [17]	46 644	–	11%
Interleukin 6 receptor subunit alpha (IL-6RA) [17]	46 643	–	8%
Soluble glycoprotein 130 (SGP130) [17]	46 643	–	8%
Transforming growth factor beta-1 proprotein (TGF-β1) [17]	46 643	–	12%
Tumor necrosis factor alpha (TNF-α) [17]	46 644	–	9%
Tumor necrosis factor receptor superfamily member 1A (TNFR1A) [17]	46 644	–	17%
Tumor necrosis factor receptor superfamily member 1B (TNFR1B) [17]	46 644	–	18%
***Immune Mediated Diseases***			
Addison’s disease (AD) [19]	1 223	4 097	16%
Celiac disease (CEL) [20]	2 364	324 074	13%
Crohn’s disease (CD) [21]	12 194	28 072	22%
Diabetes type 1 (T1D) [22]	18 942	501 638	14%
Juvenile idiopathic arthritis (JIA) [23]	3 305	9 196	13%
Myasthenia gravis (MG) [24]	1 873	36 370	11%
Psoriasis (PSO) [25]	15 967	28 194	15%
Psoriatic arthritis (PSA) [26]	5 065	21 286	13%
Rheumatoid arthritis (RA) [27]	14 361	43 923	9%
Systemic lupus erythematosus (SLE) [28]	4 036	6 959	10%
Ulcerative colitis (UC) [21]	12 366	33 609	15%
***Psychiatric Disorders***			
Alcohol use disorder (ALCH) [29]	8 485	20 272	14%
Anorexia nervosa (AN) [30]	16 992	55 525	16%
Anxiety disorder (ANX) [31]	31 977	82 114	23%
Attention-deficit/hyperactivity disorder (ADHD) [32]	38 691	186 843	18%
Autism spectrum disorder (ASD) [33]	18 381	27 969	12%
Bipolar disorder (BIP) [34]	56 287	781 022	12%
Cannabis use disorder (CUD) [35]	14 808	343 726	19%
Major depressive disorder (MDD) [36]	412 305	1 588 397	7%
Obsessive compulsive disorder (OCD) [37]	2 688	7 037	30%
Opioid use disorder (OUD) [38]	3 211	11 589	19%
Post traumatic stress disorder (PTSD) [39]	141 479	1 113 329	5%
Schizophrenia (SCZ) [40]	53 386	77 258	22%
Tourette’s syndrome (TS) [41]	4 819	9 488	22%

*Note*. This table describes all GWAS summary statistics of all traits included in the analyses. Traits where only a sample size for cases, but not controls is given, indicate that this trait had a continuous N. h^2^ represents the liability scale heritability.

**Fig 1:**
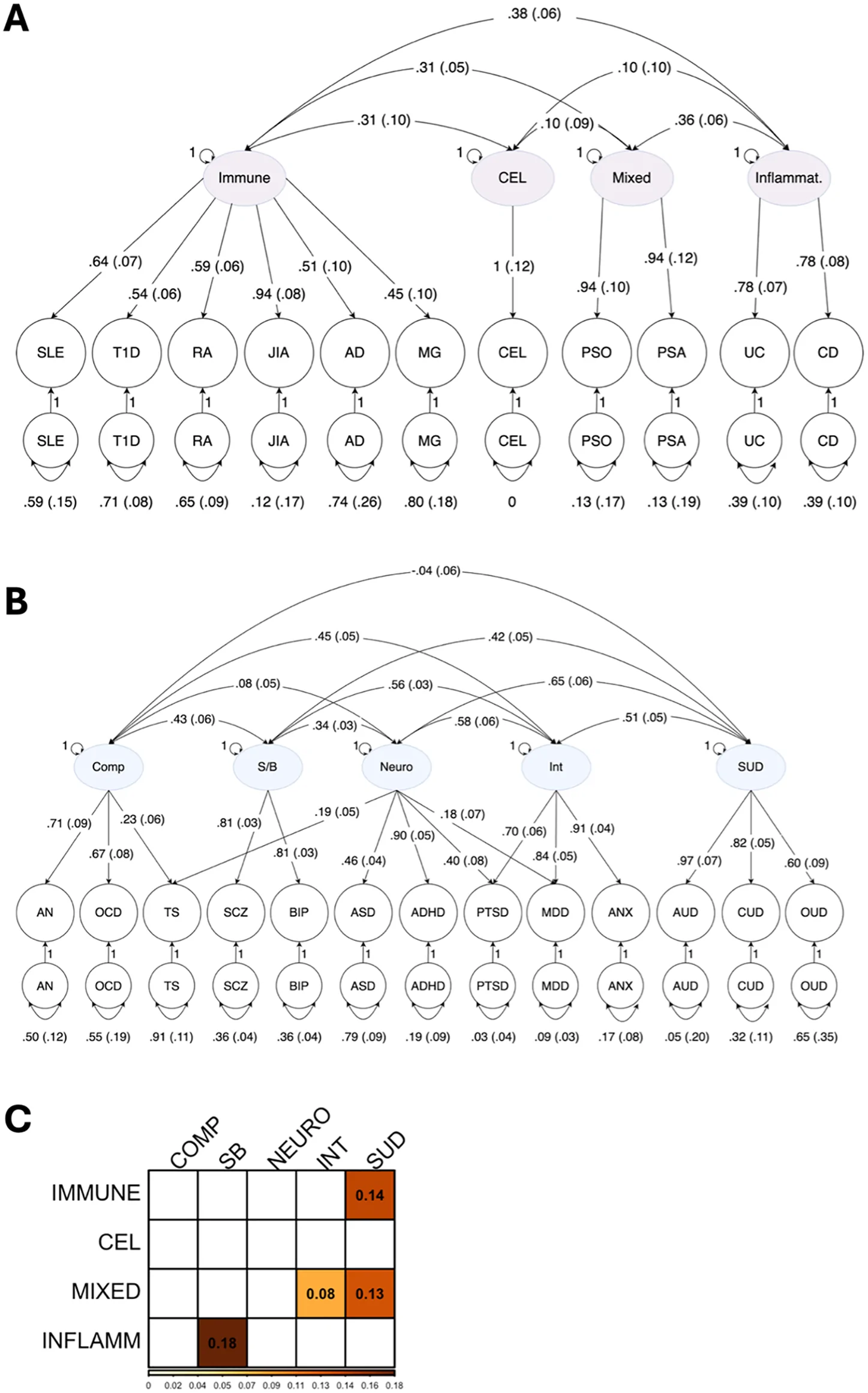
Immune-mediated disease and psychiatric disorders correlated factors models and correlations between immune and psychiatric factors. **A)** Immune-mediated diseases path diagram with standardized estimates for the correlated factors model used in genomic structural equation modelling (genomic SEM) with four factors of immune-mediated diseases. Latent variables are represented as circles. The genetic component of each phenotype is represented with a circle because the genetic component is a latent variable that is not directly measured but is inferred using linkage disequilibrium score regression (LDSC). Single-headed arrows are factor loadings; double-headed arrows connecting back to the same origin are variances; and double-headed arrows connecting two variables are correlations. Paths labeled 1 are fixed to 1; all estimates are given next to the path with standard errors in parentheses. Disease abbreviations are defined in Table 1. **B)** Psychiatric disorders path diagram. Disorder abbreviations are defined in Table 1. **C)** Genetic correlations between factors of immune-mediated diseases and factors of psychiatric disorders. IMMUNE, autoimmune diseases; CEL, celiac disease; MIXED, mixed immune pattern diseases; INFLAMM, inflammatory diseases; COMP, compulsive disorders; SB, schizophrenia/bipolar; NEURO, neurodevelopmental disorders; INT, internalizing disorders; SUD, substance use disorders.

The correlations across the psychiatric and immune factors were then estimated. Significance was defined using an FDR corrected threshold. We only considered inter-factor correlations significant when they were not also FDR significant for the *Q*_*Factor*_ heterogeneity statistic, which was used as a quality control test to evaluate whether these factor correlations accurately captured genetic overlap [4]. Non-significant *Q*_*Factor*_ results suggest that the factor correlation appropriately captures risk sharing across these two clusters of disease traits. Conversely, *Q*_*Factor*_ would be expected to be significant if disorders loading on Factor 1 had a mixture of positive and negative genetic correlations with the disorders on Factor 2, such that a single correlation between Factor 1 and 2 is insufficient for describing the cross-disorder patterning of associations. Pruning out *Q*_*Factor*_ significant associations yielded four significant inter-factor associations between immune-mediated and psychiatric diseases (S1 Table). These reflected correlations between autoimmune disease and substance use disorder factors, mixed immune pattern diseases with internalizing and substance use disorders factors, and inflammatory disorders with the schizophrenia/bipolar factor.

### Associations with immune markers

We next examined the genetic correlations between the 14 immune markers and the five psychiatric and four immune factors. We again used the *Q*_*Factor*_ heterogeneity statistic to evaluate whether genetic correlations with the markers were likely to operate at the factor level. In instances where there were significant *Q*_*Factor*_ results, we evaluated whether the immune marker was significantly associated with any of the individual disorders that defined the psychiatric or immune factor. After pruning out significant *Q*_*Factor*_ results, we identified 20 FDR significant immune marker – immune disease relationships, which included associations of ten markers with three immune-disease factors (all but the celiac factor; S2 Table). Interferon gamma (IFNG) was the strongest associated marker for the inflammatory (*r*_*g*_ = .48, standard error [*SE*] =.10) and mixed immune pattern factors (*r*_*g*_ = .35, *SE* = .09), while TNF-α had the largest correlation for the immune factor (*r*_*g*_ = .51, *SE* = .08). For individual disease associations investigated due to significant *Q*_*Factor*_ results, we observed significant correlations between Crohn’s disease and CRP (*r*_*g*_ = .16, *SE* = .03) and IL-6 (*r*_*g*_ = .33, *SE* = .06), along with ulcerative colitis and IL-6 (*r*_*g*_ = .12, *SE* = .06).

For the psychiatric factors, we identified 27 significant immune marker genetic correlations that included ten markers and four of the five psychiatric disorders factors (all but the schizophrenia/bipolar factor; S3 Table), along with five significant correlations with ADHD. Correlations were all positive, with the exception of large negative genetic correlations (range: -.21 to -.41) for 9 markers and the compulsive factor. Interleukin-1 receptor antagonist (IL-1RA) had the largest point estimate across markers for the compulsive (*r*_*g*_ = -.41, *SE* = .07) and internalizing factors (*r*_*g*_ = .26, *SE* = .04) and for ADHD (*r*_*g*_ = .35, *SE* = .04). C-C motif chemokine 3 (CCL3) had the largest correlation for the neurodevelopmental (*r*_*g*_ = .23, *SE* = .05) and substance use factors (*r*_*g*_ = .32, *SE* = .07).

Two pairs of markers, tumor necrosis factor receptor superfamily member 1A and B (TNFR1A & TNFR1B) and tumor necrosis factor (TNF) and C-C motif chemokine 3 (CCL3), displayed highly similar patterns of genetic correlations across psychiatric and immune factors. Both marker pairs additionally consisted of highly genetically correlated (see S1 Fig) and mechanistically related markers in terms of their function within the immune system. TNFR1A and B are both representatives of the same TNF-receptor superfamily and part of the same signaling network that influences the effect of TNF-α on macrophage viability and are closely integrated with pattern recognition receptor regulated signaling systems [43]. Similarly, TNF-α and CCL3 are part of the same signaling pathway, with TNF-α directly promoting CCL3 production [44]. Consequently, these two marker pairs were each modeled as two-indicator factors in subsequent mediation analyses to reflect the joint genetic architecture and previously described biology that they share.

To evaluate whether our immune marker results were robust across datasets, replication analyses were conducted for the four immune markers that had separate, well-powered GWAS available. Due to the limited number of available markers with external GWAS, these results are primarily detailed in the Supplementary Results section of S1 Text. We note here that 100% of the associations of psychiatric disorders and immune-mediated diseases with CRP, 67% of associations with IL-1RA, 56% of associations with TNF-α, and 13% of associations with CCL3 replicated in the external dataset in terms of significance and direction of effect. This gradient in overlap closely mirrors the discrepancy in power of the respective markers’ replication dataset with that used in the original analyses.

### Multiple regression and Mendelian randomization reveal null effects of CRP

In line with prior work describing links between CRP and psychiatric conditions, significant correlations were found for the compulsive (*r*_*g*_ = -.37, *SE* = .05), internalizing (*r*_*g*_ = .13, *SE* = .02) and substance use disorder (*r*_*g*_ = .23, *SE* = .04) factors and for ADHD (*r*_*g*_ = .30, *SE* = .02). As IL-6 stimulates CRP production [45], it is unsurprising that IL-6 showed a highly similar pattern of associations, again with the compulsive (*r*_*g*_ = -.35, *SE* = .08), internalizing (*r*_*g*_ = .23, *SE* = .04) and substance use disorder (*r*_*g*_ = .29, *SE* = .07) factors and ADHD (*r*_*g*_ = .34, *SE* = .05). Since CRP is produced by the liver and not able to cross the blood-brain-barrier (despite altering its permeability) [46], and it is biologically more likely that IL-6 that can cross the blood-brain barrier [47], it is plausible that CRP was merely correlated with psychiatric disorders due to its relationship with IL-6. To investigate this, we fit multiple regression models with CRP and IL-6 as correlated predictors to test the partial effects of each marker on the psychiatric disorders or immune diseases (S2 Fig). These revealed that only IL-6 continued to have significant partial associations (S4 Table). This finding was replicated in multiple regressions including an independent CRP GWAS dataset and the same IL-6 GWAS dataset (due to the lack of a sufficiently powered independent IL-6 GWAS dataset S1 Text).

Additionally, bidirectional Mendelian randomization (MR) between CRP and IL-6 supported by Steiger directionality testing, confirmed a highly significant, reciprocal relationship between IL-6 and CRP (p_Steiger_IL6-CRP_ = 2.57e-80; p_Steiger_CRP-IL6_ = 1.01e-39). Notably, this test was much more significant for the IL-6 on CRP direction, despite the much larger sample size of CRP, and the larger number of instruments that was identified for this biomarker (N_SNP_CRP_ = 234; N_SNP_IL6_ = 5). The absolute effects were also larger for IL-6 than for CRP, but inconsistencies in significance across the MR-Egger (β_IL6-CRP_ = -.69; p_IL6-CRP_ = 2.11e-02; β_CRP-IL6_ = -.34; p_CRP-IL6_ = 1.94e-05) and the weighted median method were observed (β_IL6-CRP_ = -.38; p_IL6-CRP_ = 3.50e-110; β_CRP-IL6_ = .06; p_CRP-IL6_ = 1.28e-01). Sensitivity analyses of the bidirectional models additionally revealed significant heterogeneity across instruments (Cochran’s Q p < 0.001) and evidence of directional horizontal pleiotropy via the MR-Egger intercept test (IL-6 intercept p = 0.045; CRP intercept p < 0.001). Consequently, the univariable effect estimates from these bidirectional models may be confounded by shared pathways and should be interpreted as capturing both direct and pleiotropic effects. While outlier removal is a common approach to address such assumption violations, discarding these variants would obscure true biological signal in this context. This observed pleiotropy is expected given the enmeshed nature of the inflammatory cascade, where genetic variants frequently influence multiple highly correlated immune markers simultaneously. Because this correlated pleiotropy violates the exclusion restriction assumptions of univariate MR, it necessitated a structural modeling solution rather than variant exclusion.

Multivariable MR (MVMR) was used to jointly model IL-6 and CRP as predictors of psychiatric outcomes. This produced partial causal effect estimates on the psychiatric outcomes that control for genetic overlap across these immune markers. In the joint genetic model predicting psychiatric disorders, genetically predicted IL-6 retained a significant direct causal effect for internalizing disorders (β_IL6-INT_ = .04; SE = .02; p = .011) and substance use disorders (β_IL6-SUD_ = .07; SE = .02; p = .002), whereas the effect of CRP was attenuated for internalizing (β_CRP-INT_ = .02; SE = .01; p = .057) and for substance use disorders (β_CRP-SUD_ = .03; SE = .02; p = .04). For ADHD neither marker was significant, although the effect estimate was larger and the p-value smaller for IL-6, indicating that this is likely a power issue. CRP was therefore dropped from all subsequent mediation models in favor of IL-6. Fig 2 shows how previously identified strong and significant effects of CRP on psychiatric disorders can be attenuated and become insignificant when accounting for shared signal with IL-6. Results for the bidirectional analyses and their sensitivity analyses are provided in S24 Table and results for the MVMR analyses and their sensitivity analyses are provided in S25 Table.

**Fig 2:**
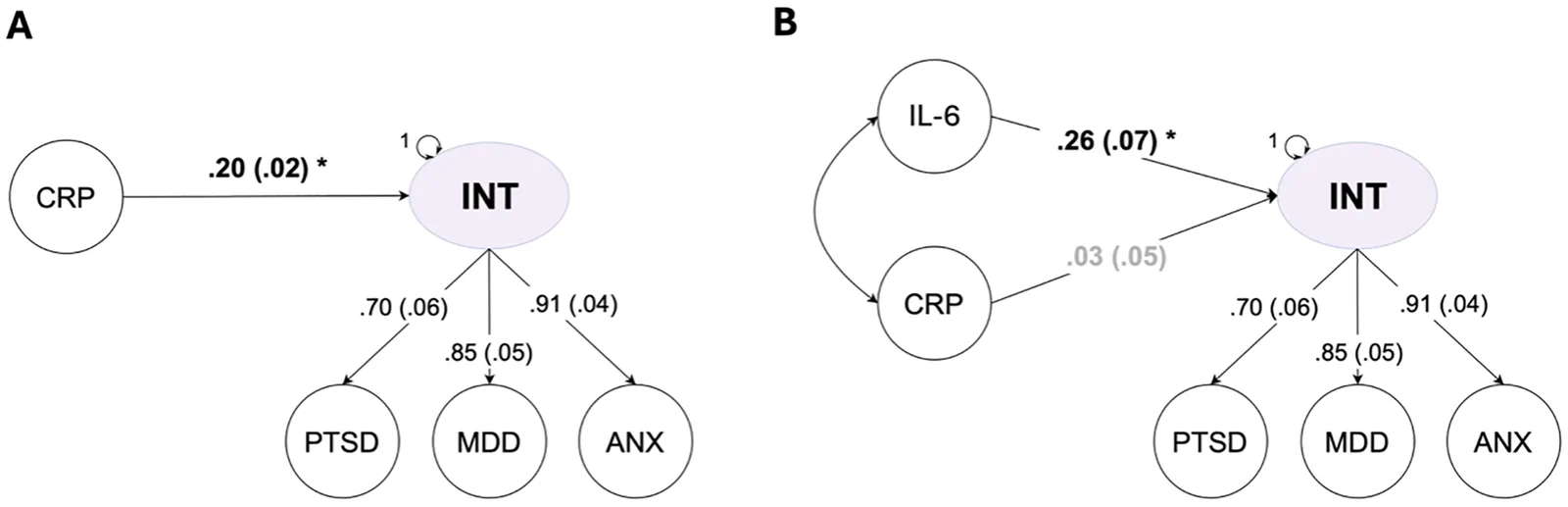
C-Reactive protein regression models. Regression model of the internalizing disorders factor regressed on CRP in Panel **A** and a multiple regression model of the internalizing disorders factor regressed on CRP and IL-6 in Panel **B**. This demonstrates how significant relationships of CRP with psychiatric disorders may be better explained by IL-6. Factor loadings of the internalizing factor differ from those in the correlated factors model (Fig 1) because cross-loadings are dropped for the follow up regression models. Single-headed arrows indicate regression relationships as modelled in genomic SEM and importantly do not imply expected or modeled directional relationships.

### Immune markers mediate immune-psychiatric associations

We aimed to produce a robust set of findings by only testing mediation models that met three requirements: (i) the marker was significantly associated with an immune disease factor or disorder (often denoted the *a* – path in a mediation model), (ii) the marker was significantly associated with a psychiatric factor or disorder (*b* – path), and (iii) the disorders or factors from the *a* and *b* paths were themselves significantly associated (the total effect to be mediated, often denoted the *c* – path). This process resulted in three types of mediation models that tested a marker’s mediation of: a *factor-factor* relationship, representing an immune-mediated disease factor to psychiatric factor relationship; a *factor-trait* relationship, representing an immune-mediated disease factor to an individual psychiatric disorder relationship or a psychiatric disorder factor to and individual immune-mediated disease relationship, or finally; a *trait-trait* relationship that represents an individual immune-mediated disease to an individual psychiatric disorder relationship.

We tested 12 mediation models for six markers that met the inclusion criteria described above (as detailed in Fig 3 with model fit statistics in S5 Table and model outputs in S6–S17 Tables). Of these 12, 11 were significant at an FDR threshold. A graphical representation of the direct, indirect and total effects of each mediation is depicted in Fig 4 and a pie chart of the proportions of these effects for each mediation model can be found in Fig 5. Six of the mediations were for *factor-factor* relationships, five were for *factor-trait* relationships. There were no *trait-trait* relationships that exhibited significant mediation by an immune marker.

**Fig 3:**
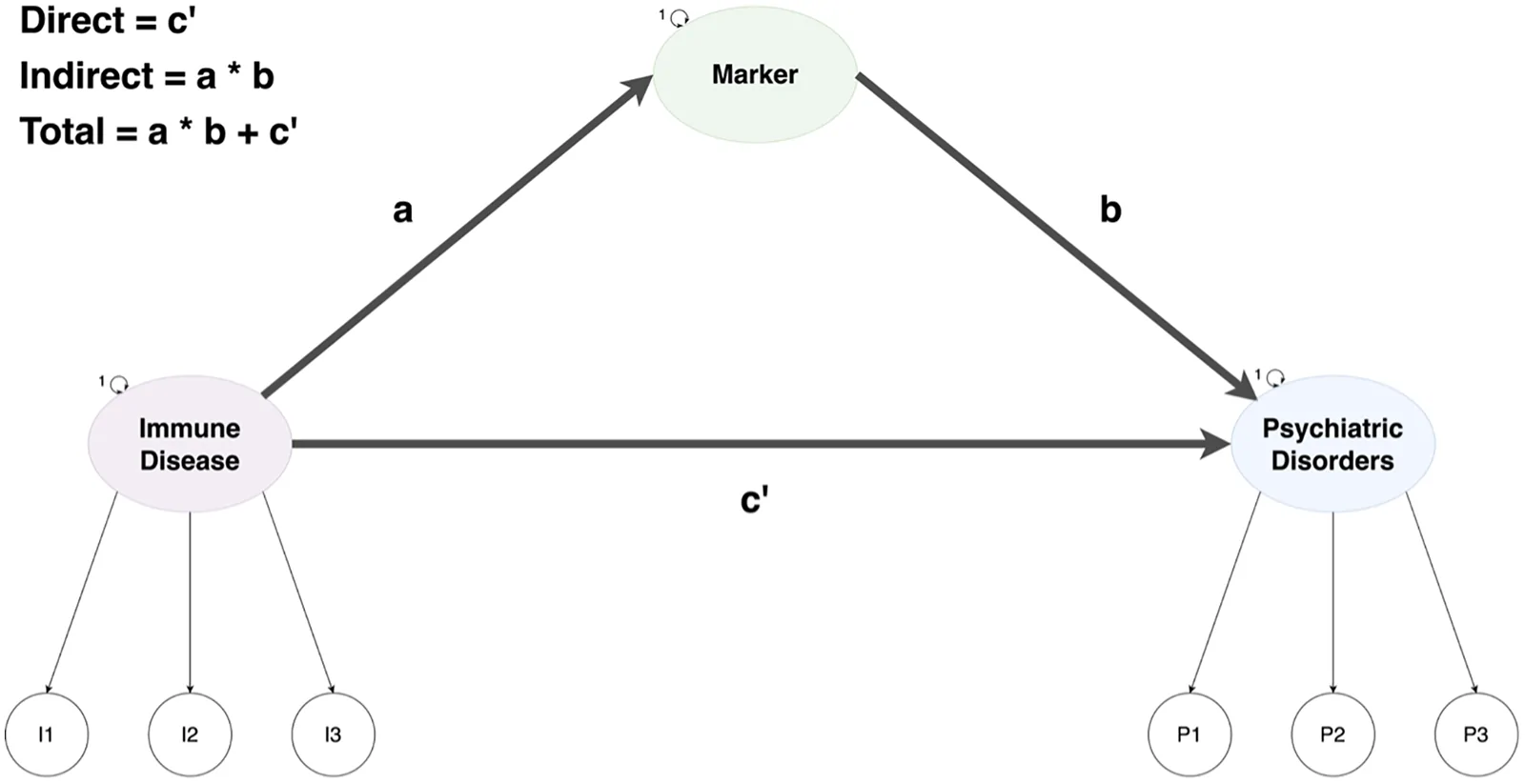
Example mediation model. Example of a mediation model for the relationship between an immune disease factor with indicators I1-3 and a psychiatric disorder factor with the indicators P1-3. The marker serves as a mediator and the corresponding *a*, *b*, and *c*’-path are tested and with them, the direct, indirect, and total effects are calculated according to the above equations. Single-headed arrows indicate regression relationships as modelled in genomic SEM and importantly do not imply expected or modeled directional relationships.

**Fig 4:**
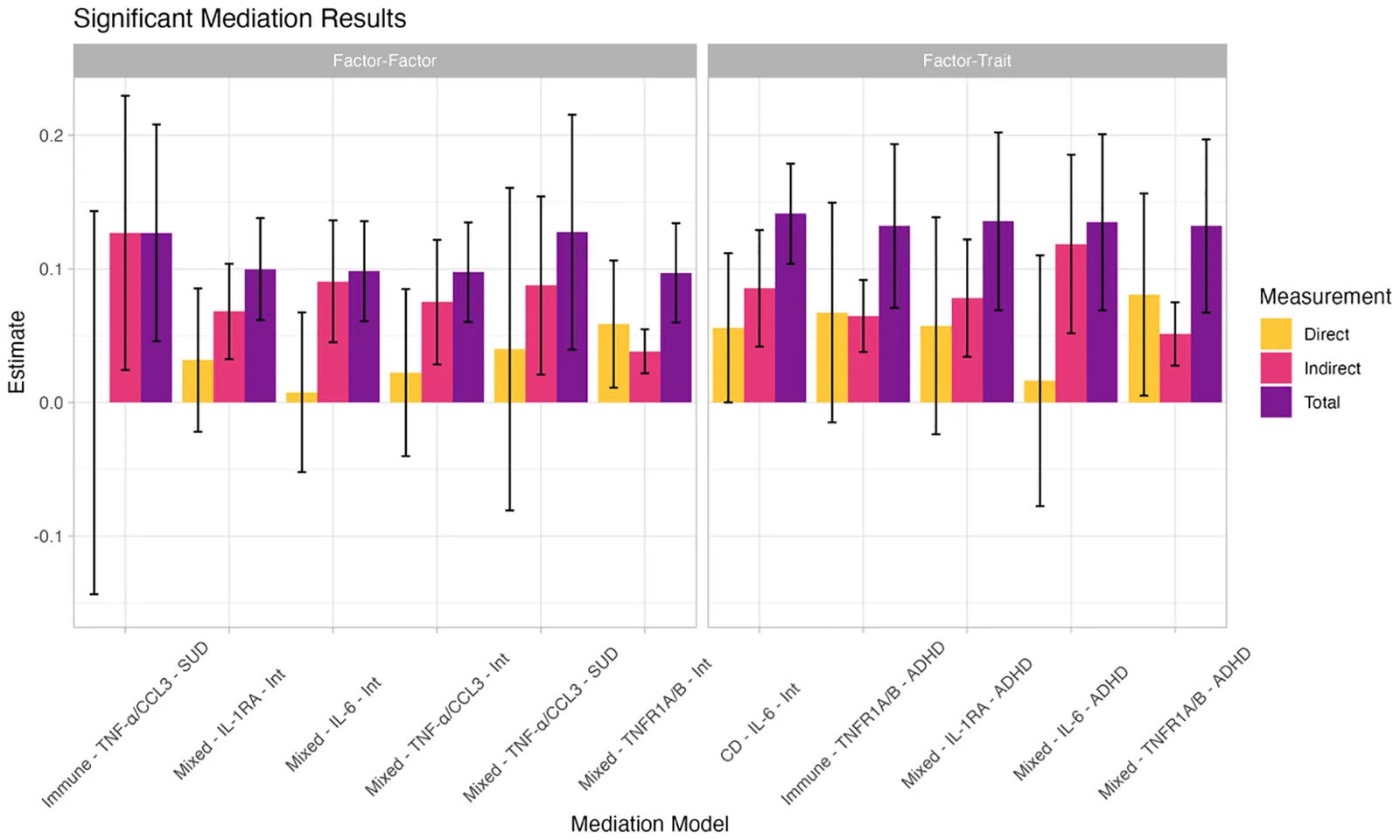
Significant mediation model effect estimates. Direct, indirect, and total effect estimates for each of the tested mediation models. Error bars display 1.96 * standard error. All mediation models displayed here were significant, as indicated by the significant indirect effect estimates and additionally, all total effect estimates were significant. Immune, autoimmune diseases; Mixed, mixed immune pattern diseases; Int, internalizing disorders; SUD, substance use disorders; CD, Crohn’s disease; ADHD, attention-deficit/hyperactivity disorder.

**Fig 5:**
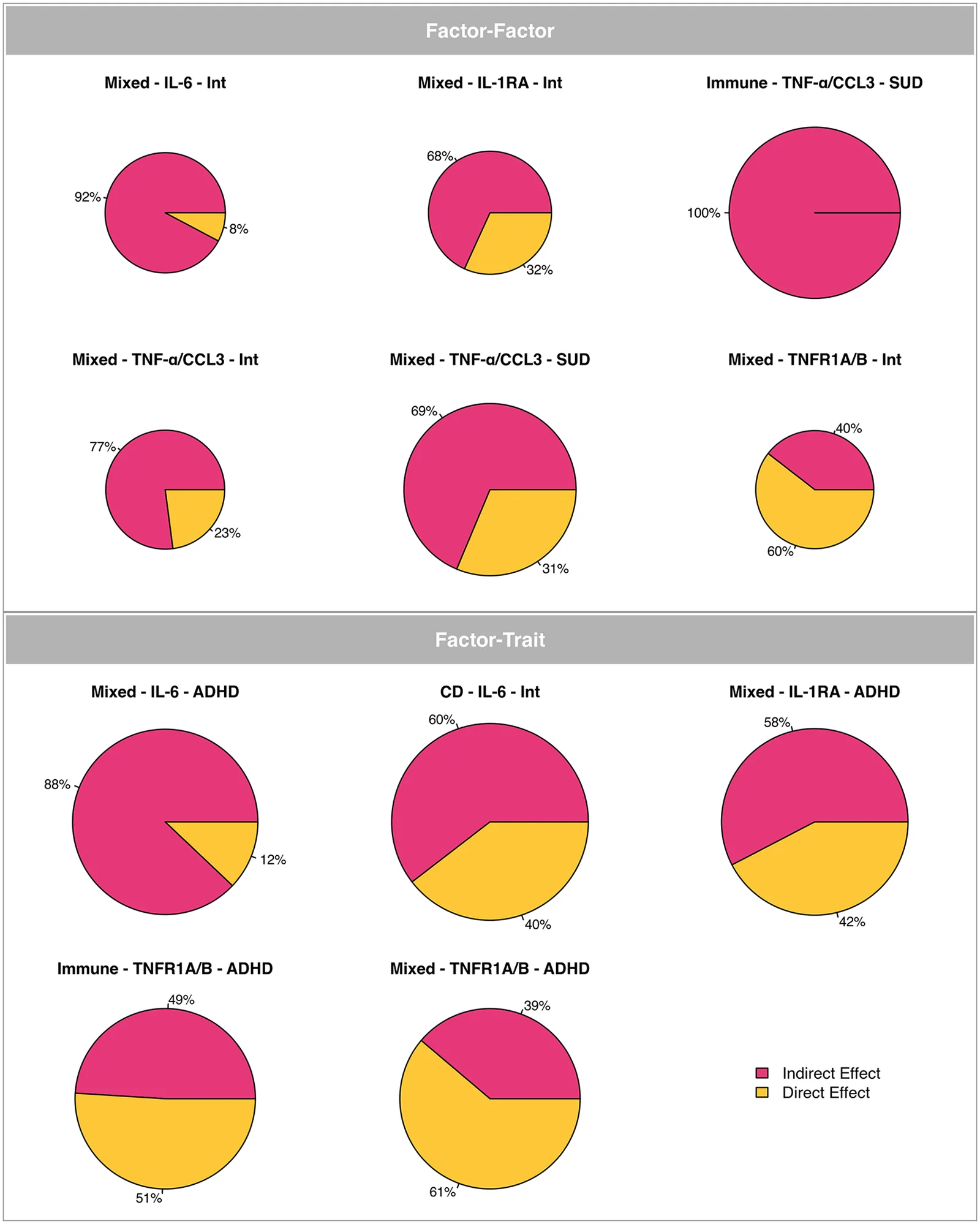
Proportions of direct and indirect effects. Pie charts are displaying the proportions of the direct and indirect effects for each of the mediation models tested. The size of the pie charts represents the size of the total effect. Immune, autoimmune diseases; Mixed, mixed immune pattern diseases; Int, internalizing disorders; SUD, substance use disorders; CD, Crohn’s disease; ADHD, attention-deficit/hyperactivity disorder. **A** displays all factor-factor relationships and **B** displays all factor-trait relationships.

Four of the six *factor-factor* models reflected mediation of the association between the mixed immune pattern and the internalizing factors by IL-6, IL-1RA, the combined TNF-α/CCL3 factor, and the TNFR1A/TNFR1B factor. The largest mediating effect was by IL-6, where the direct effect was attenuated to only.01 (*SE* = .03), and the indirect effect was.09 (*SE* = .02, *p*_*FDR*_ = 2.98e-04), such that IL-6 mediated ~92% of the total effect. The largest effect across models was for TNF-α/CCL3, which mediated 100% of the association between a substance use and autoimmune factor, wherein the direct effect was estimated at 0 (*SE* = .07), and the indirect effect at.13 (*SE* = .05, *p*_*FDR*_ = 1.96e-02).

Within the *factor-trait* mediation models, we found that IL-6 mediated ~60% of the relationship between the internalizing factor and Crohn’s disease, with a direct effect of.05 (*SE* = .02) and indirect effect of.09 (*SE* = .02, *p*_*FDR*_ = 2.98e-04). Similar to the large number of mediation between mixed immune pattern diseases and internalizing disorders, three factor-trait mediation models were identified for the mixed immune pattern factor and ADHD, with the markers reflecting IL-1RA, TNFR1A/TNFR1B, and IL-6. The marker that again accounted for the largest proportion of the total effect was IL-6, with a direct effect of only.02 (*SE* = .05) and an indirect effect of.12 (*SE* = .03, *p*_*FDR*_ = 7.97e-04) that accounted for ~88% of the total effect.

### Multiple mediator models

The relationships between mixed immune pattern diseases and both internalizing disorders and ADHD were significantly mediated by the genetic liability for several immune markers, which together accounted for more than 100% of the total effect. This, along with the fact that these markers were themselves highly genetically correlated (S1 Fig), indicated that the mediating markers were tapping into interrelated biological pathways. This prompted running multiple mediator models that included the set of identified markers as correlated mediators (Fig 6). For the mixed immune pattern disease and the internalizing factor, the set of markers (IL-6, IL-1RA, TNF-α/CCL3, TNFR1A/B) collectively mediated 100% of the relationship, with a direct effect estimated at 0 (*SE* = .03, *p*_*FDR*_ = 9.56e-01) and a significant collective indirect effect of all markers combined of.10 (*SE* = .02, *p*_*FDR*_ = 2.79e-05). Only the individual mediating effect of IL-1RA remained significant with an indirect effect of.05 (*SE* = .02, *p*_*FDR*_ = 1.75e-02). The multiple mediator model for ADHD by IL-1RA, IL-6, and TNFR1A/B revealed a non-significant direct effect (.01; *SE* = .05, *p*_*FDR*_ = 8.84e-01) and a significant cumulative indirect effect of all markers combined of.13 (*SE* = .03, *p*_*FDR*_ = 5.44e-06) that accounted for ~94% of the total effect. We were not sufficiently powered to detect significant partial mediating effects of any markers.

**Fig 6:**
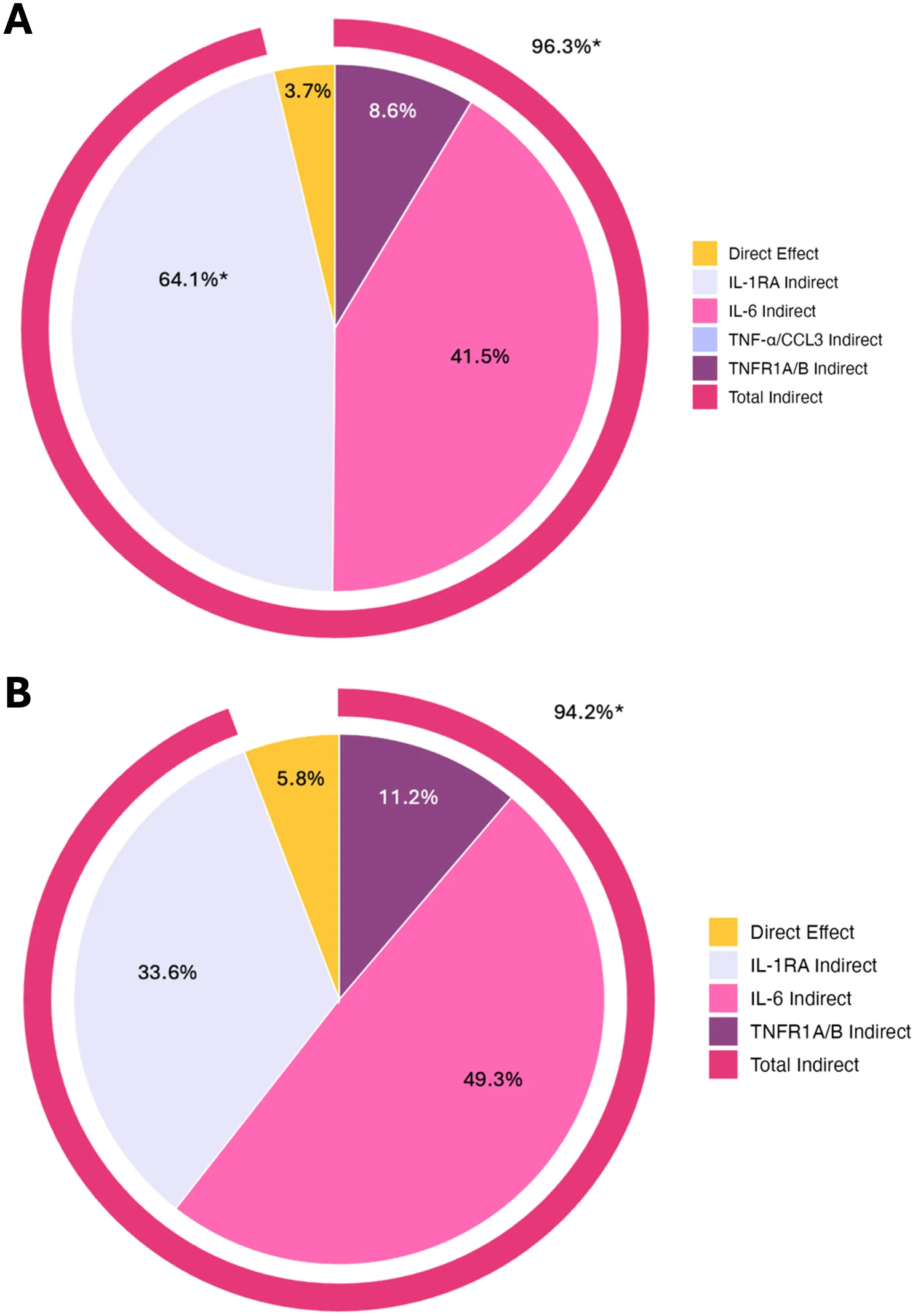
Multiple mediator model results. Pie charts are displaying the proportions of the direct and indirect effects, with the total indirect effect broken down into the effects of the individual markers in a multiple mediation framework. Immune, autoimmune diseases; Mixed, mixed immune pattern diseases; Int, internalizing disorders; SUD, substance use disorders; CD, Crohn’s disease; ADHD, attention-deficit/hyperactivity disorder. **A** Multiple mediation results for the mixed immune pattern disease and internalizing disorder relationship. **B** Multiple mediation results for the mixed immune pattern disease and ADHD relationship.

## Discussion

Epidemiological and genetic work has described substantial relationships between immune-mediated diseases and psychiatric disorders. In the current study, we provide evidence that these genetic relationships are mediated by immune and inflammatory biomarkers. This represents a systematic and comprehensive multivariate solution to characterize the large number of bivariate associations between immune-mediated and psychiatric diseases and immune markers. Our results clarify which of the genetic links between immune-mediated diseases and psychiatric disorders are mediated by the genetic liability for immune markers and which markers mediate the relationships (and which markers do not). Additionally, heterogeneity analyses identified which immune-psychiatric associations were better described at the level of individual disorders (such as ADHD with mixed immune pattern diseases) as opposed to more transdiagnostic shared pathways captured across, for example, the mixed immune pattern diseases and internalizing disorders factors.

Our analysis highlights the mediating roles of IL-6, IL-1RA, and the shared biology across the TNF-α/CCL3 and TNFR1A/B factor. Notably, IL-6 significantly mediated the majority of three psychiatric-immune relationships: 92% of the association between internalizing and mixed immune pattern disease, 88% between ADHD and mixed immune pattern disease, and 60% of the genetic link between internalizing disorders and Crohn’s disease. The latter finding is consistent with prior conceptualizations of IL-6 as the inflammation-based mechanism underlying the association, which can lead from internalizing disorders to inflammatory bowel disease [48] and vice versa [49]. The broader set of associations for IL-6 suggest that it may confer more transdiagnostic risk beyond just the internalizing and Crohn’s relationship. At the same time, the TNF-α/CCL3 markers were found to significantly mediate 100% of the relationship between substance use disorders and autoimmune diseases and TNFR1A/B were the only markers that mediated the immune and ADHD association. In addition, the multiple mediator models for the mixed immune pattern and internalizing factors and ADHD revealed that the cumulative mediating effect across all markers was greater than the individual effect of all markers. These results collectively indicate the importance of investigating multiple markers for psychiatric-immune relationships, though our results also help narrow the search space by identifying unrelated markers.

The specific mapping of these immune pathways onto distinct cross-disorder patterns aligns closely with current immunopsychiatric models of central nervous system (CNS) dysfunction. The prominent transdiagnostic role of IL-6 and IL-1RA in mediating the shared variance between mixed immune pattern diseases, internalizing disorders, and ADHD likely reflects their well-documented capacity to breach the blood-brain barrier and modulate neurocircuitry governing affect and executive function. This is consistent with IL-6’s documented effects on tryptophan metabolism and corticolimbic structure relevant to internalizing phenotypes [50], and on dopaminergic transmission relevant to executive function in ADHD [51]. The mediating effect of IL-1RA, a competitive antagonist of IL-1β, suggests that regulation of the inflammatory response is as relevant to psychiatric risk as the pro-inflammatory signal itself [52].

In contrast, the distinct finding that the TNF-α/CCL3 and TNFR1A/B pathways specifically mediate the relationship between autoimmune diseases and substance use disorders (SUD) points toward a different pathophysiological mechanism centered on mesolimbic reward circuitry. TNF-α and CCL3 are potent drivers of cellular trafficking and neurovascular inflammation [53] and have been linked to synaptic remodeling in the nucleus accumbens that dampens reward sensitivity and promotes behavioral transitions associated with substance dependence [54,55]. By demonstrating that these independent immune signaling networks selectively map onto distinct symptom dimensions like affective and executive circuits for IL-6/IL-1RA versus reward and plastic dependency pathways for TNF-α/CCL3, our genomic factor-specific findings provide empirical support for a highly differentiated model of immunopsychiatric risk.

In addition to the significant associations observed, it is relevant to consider the patterns of null results, particularly given a literature that describes such widespread associations. For example, while our study links psychiatric disorders to IL-1β and TGFβ-1, they did not mediate any of the psychiatric-immune relationships. Furthermore, the included markers did not mediate the association between inflammatory diseases and the schizophrenia/bipolar factor, even though markers that were included here (like CRP and IL-6) have been previously associated with both disorders [56,57]. This likely reflects methodological differences, rather than true inconsistencies in findings. Prior genetic studies linking schizophrenia and bipolar disorder to markers like CRP and IL-6 frequently utilize MR, which relies on a restricted set of strong genetic instruments as proxy for circulating biomarker levels and test for directional effects. By contrast, the genetic correlations and Genomic SEM models estimated here evaluate the highly polygenic, genome-wide shared architecture between traits. Specific, localized genetic instruments can exert significant causal effects in the absence of a significant, genome-wide genetic correlation [58].

The most notable set of null results was for CRP. This is notable given a prior literature wherein CRP has been extensively associated with immune-mediated diseases and psychiatric disorders [59–61]. Additionally, CRP has been found to have overlapping genetic loci with those of psychiatric disorders [62], with authors going so far as to suggest using CRP as a biomarker for diagnosing the presence and severity of psychiatric disorders [63]. The current findings revealed that the effect of CRP on immune-mediated diseases and psychiatric disorders was highly attenuated and nonsignificant when IL-6 was included as a genetically correlated predictor. The central role of IL-6 was supported by MVMR where IL-6 maintained a stronger and more significant causal effect than CRP in a joint genetic model predicting the psychiatric disorder. These findings are consistent with biological evidence and other literature looking at depression outcomes and including both markers [64]. CRP cannot cross the blood-brain-barrier (despite altering its permeability) [46] while IL-6 can [47], therefore making it more likely that IL-6 could have more direct links to psychiatric disorders whose genetic risk is concentrated in the brain [45,65]. This finding more generally highlights the critical importance of comprehensive, multivariate analyses going forward that include multiple correlated markers of the immune system. Furthermore, it emphasizes that the specific statistical architecture applied, such as partial-effect testing for sequential biological cascades versus latent factor modeling for complementary pathways, must be carefully tailored to the distinct physiological roles of the markers involved.

The findings from this study may have clinical and translational implications, particularly concerning biomarker prioritization and therapeutic intervention. One impact of delineating these specific genetic architectures lies in the potential for drug repurposing. Identifying the precise immune markers that mediate the relationship between immune and psychiatric phenotypes provides a targeted roadmap for evaluating existing immune-based therapies, some of which have already demonstrated efficacy in treating subsets of depression [66]. If downstream causal analyses confirm directional pathways between these specific immune markers and psychiatric disorders, there is strong potential for integrating these markers into clinical screening efforts. Longitudinal tracking of prioritized biomarkers via electronic health records could ultimately facilitate earlier detection and more personalized, mechanistically targeted interventions for psychiatric disorders.

GWAS data was limited to individuals of European-like ancestry since it was only of sufficient sample size in this ancestral group. Future work should extend these analyses to other ancestral groups once sample sizes are sufficient. Furthermore, GWAS summary statistics included in this study remove the major histocompatibility complex (MHC) because of its complex LD-structure [67], driven in part by segmental duplications, structurally variant loci (e.g., *C4*, *DRB*), and duplicated repeat elements that generate recombinant haplotypes [68]. However, the human leukocyte antigen gene (HLA) [69] in the MHC region codes for genes that are relevant for immune function, and we are therefore missing genetic links that are driven by this region of the genome. Recent methods quantifying genetic correlations based on convergence across haplotype group-disease associations in the HLA find that for many diseases pairs the HLA block relationships did not match up with the overall genetic correlations of the rest of the genome [70]. Future work should assess this for the included immune diseases and psychiatric disorders as well as the immune markers. Another region that is removed from GWAS summary statistics because of the difficulties in analyzing them are the sex chromosomes. This exclusion is significant in the context of our study, since the X chromosome contains a high density of immune-related genes and regulatory elements that are involved in the innate and adaptive immune responses [71,72]. Sex differences are well documented in both autoimmune disease prevalence [60,73] and several psychiatric disorders [74], including a proposed protective effect of X-linked gene dosage in autism [75]. Therefore, future research would benefit from explicitly analyzing the sex chromosomes or considering sex-specific genetic links between autoimmune disease and psychiatric disorders. Additionally, GWAS for biomarkers may include genetic signal for behavioral traits (such as smoking) that influence biomarker levels. While our framework statistically accounts for sample overlap, the heavy reliance on minimally phenotyped cohorts introduces ascertainment biases that can lower clinical specificity [76] and artificially attenuate genetic correlations when modeled alongside clinical cohorts [77]. Despite displaying the mediation models with directional, single-headed arrows and specifying the models in terms of regression relationships, causal relationships between immune mediated diseases, psychiatric disorders, and immune markers cannot be inferred from our models. They are displayed in this way to be consistent with how they are modelled within genomic SEM, but the values obtained from the models would be the same if the arrows were reversed.

By systematically modeling the genetic liability for 14 immune markers across a broad set of immune-mediated diseases and psychiatric disorders, this study moves beyond prior bivariate associations to identify which specific markers account for shared genetic risk, and for which disorder pairs. Six markers emerged as significant mediators, with particularly large and pervasive effects observed for IL-6, though which markers were most relevant was specific to the disorders included. This indicates that immune-psychiatric comorbidity reflects multiple, partially distinct biological pathways rather than a single shared mechanism. These findings were produced from genetic correlations and generally establish shared genetic architecture rather than confirmed causal pathways. Potential clinical translation will require extensive validation in longitudinal and experimental designs, though our current MR findings that are consistent with causal effects of the immune markers on psychiatric condition indicate that this is a promising area for future investigation. By pinpointing which immune pathways are most relevant to which psychiatric phenotypes, this work provides a foundation for prioritizing future mechanistic and translational research into immune-psychiatric comorbidity.

## Methods

### Phenotype selection and quality control

Recent studies of markers of the immune system associated with psychiatric disorders were referenced to compile an initial list of immune markers. The literature across psychiatric disorders commonly linked to immune markers was referenced [59,60,78,79], and cross referenced with the list of available circulating metabolites (https://olink.com/products/olink-explore-3072-384). This approach was chosen over a more comprehensive approach to narrow down the large number of immune and inflammatory markers to avoid excessive multiple testing correction. This ensured that meaningful biological pathways in the context of immune mediated diseases and psychiatric disorders could be identified. Immune markers that were identified as important in the context of psychiatric disorders but do not have an associated GWAS due to their relatively invasive assessment and therefore small sample sizes (such as translocator protein, which is assessed through Positron Emission Tomography (PET) scans) were excluded. The initial list of immune markers was then filtered based on availability of sufficiently powered (reflected by a SNP-based heritability ($h_{SNP}^{\text{2}}$) *Z*-statistic > 4) [80], publicly available genome-wide association study (GWAS) summary statistics (excluding for example Interleukin-2 due to low power). For each phenotype, we chose the most well-powered, publicly available GWAS conducted on unrelated individuals of European-like genetic ancestry. We excluded traits with GWAS summary statistics that had low genomic coverage (fewer than 850,000 SNPs), which led to a final selection of 14 immune markers. For psychiatric and immune-mediated disease traits, the same GWAS summary statistics of 13 disorders [42] and 11 immune mediated diseases [4] from their previously established factor structures were used respectively. In the case of the psychiatric disorders, the factor structure from the original paper was retained, while GWAS summary statistics that had a more recent version with larger sample sizes since the publication, were updated in the model. These included PTSD that increased in the total sample size (including cases and controls) from 9,537–1,254,808, bipolar disorder that increased from 413,466–840,309, and MDD with a sample size increase from 500,199–2,009,702. All GWAS summary statistics were subjected to a standardized quality control procedure using the “munge” function from the GenomicSEM R package. This function limits the data to HapMap3 SNPs, standardizes the reference alleles across all GWAS summary statistics, and applies filters for a minor allele frequency greater than 1% and imputation quality above 0.9, where available. All GWAS summary statistics and their sample characteristics are presented in Table 1.

### LD-score regression

We performed multivariable Linkage Disequilibrium (LD)-score regression (LDSC) [80] using LD scores calculated from the European-like genetic ancestry subset of the 1000 Genomes Phase 3 project. The MHC region was excluded due to its complex LD structure, which could bias the results [67]. Multivariable LDSC generates two matrices: (*i*) a genetic covariance matrix with SNP-based heritabilities on the diagonal and genetic covariances on the off-diagonal, and (*ii*) a sampling covariance matrix with squared standard errors of the estimates and sampling dependencies representing participant sample overlap on the diagonal and off-diagonal, respectively. For binary traits, such as disease or disorder status, the LDSC estimates were converted to a more interpretable liability scale, which accounts for the continuous distribution of genetic risk (liability) and corrects for sample ascertainment. The conversion to the liability scale was based on population prevalence from the corresponding GWAS paper or a representative prevalence for the study sample. Additionally, we used the sum of effective sample sizes from the contributing cohorts for the ascertainment correction, to produce the accurate estimates of the heritability on the liability-scale [81]. For these analyses, we entered a sample prevalence of 0.5 in LDSC [81], as the sample ascertainment had already been corrected through the effective sample size calculation. For continuous traits, such as the immune markers, the total continuous sample sizes were used, and no corrections were necessary.

### Genomic SEM

We utilized genomic SEM to model the genetic overlap across immune markers and subsequently conduct mediation analyses with the genetic liability for immune markers mediating the genetic relationships between immune-mediated diseases and psychiatric disorders. Genomic SEM takes as input the output from multivariable LDSC along with a user specified SEM [82]. Good model fit was established based on the comparative fit index (CFI ≥ .9) and the standardized root mean squared residual (SRMR ≤ .1). To account for varying levels of sample overlap and differing sample sizes and therefore precision of our included traits, we utilized genomic SEM’s default diagonally weighted least squares estimator.

To determine the genetic factor structure of immune markers, we ran exploratory factor analyses (EFA) utilizing genomic E-SEM [83]. We used this to specify a confirmatory factor analysis where markers loaded on a factor when they had a standardized factor loading > .4. This cutoff is flexible and on the higher end of cutoffs commonly used [84], reflecting the fact that higher loadings might be expected given the biologically proximate nature of a set of immune markers. The best fitting model was a three-factor solution that fit the data well (CFI = .959, SRMR = .071); however, this model excluded four of the markers that did not load onto any of the factors, and among the remaining 10 markers, the identified model was overly complex with many markers cross-loading on multiple factors. To facilitate interpretation, individual markers of the immune system were therefore used as mediators of the relationships between immune disease and psychiatric disorders. However, rather than clustering markers purely on their genetic overlap with each other, we also adopted a more outcome-oriented approach for markers within the same theoretical immune pathways. Because there were clear patterns in how certain markers relate to the immune diseases and psychiatric disorders in the following *a-* and *b-*path analyses, TNF-α/CCL3 and TNFR1A/B were combined into two-indicator factors. This strategy allowed us to account for shared biological mechanisms while maintaining interpretable association patterns with the outcomes.

Before running the mediation models, we first tested whether the individual associations within the mediation model, denoted the *a*, *b*, and *c* path, were significant. The *a*, *b*, and *c* paths represent the immune-mediated disease factor to marker relationship, the psychiatric disorder to marker relationship, and the immune-mediated disease to psychiatric disorder relationship respectively. This filtering step was implemented to reduce false positives and increase power to identify true biological signal after multiple testing correction. While this approach might miss significant mediation where the indirect effect masks a significant direct effect (yielding a nonsignificant total effect), it ensures that our analysis isolates viable biological signals and identifies meaningful biological pathways. The psychiatric-immune factor correlations (i.e., the c paths) were estimated in Genomic SEMs that included the full set of psychiatric and immune factors and multiple testing correction was applied to evaluate significance (model specification given in S1 Text). Next, the five psychiatric and four immune disease factors were specified to correlate with all immune markers in two separate models. This was set up similar to the immune-psychiatric factor relationships with the model for the *a*-path specifying correlations between the markers and the immune diseases and the model for the *b*-path specifying correlations between the markers and the psychiatric disorders (model specifications given in S1 Text). Significant correlations between immune disease factors and the genetic liability for immune markers as well as between the genetic liability for immune markers and psychiatric disorders factors denote significant *a* and *b* paths of the mediation model respectively. The *a* and *b* paths were considered as being significant if they surpassed the FDR-corrected significance threshold of 14x5 and 14x4 trait-marker combinations and if those relationships did not at the same time become *Q*_*Factor*_ significant. *Q*_*Factor*_ is a measure of local model misfit, specific to a pair of factors, that captures inter-factor correlations which fail to accurately reflect the genetic relationships between the indicators (in our case psychiatric disorders or immune mediated diseases) that define these factors [4]. When the correlation is significant and *Q*_*Factor*_ is non-significant, it suggests that the factor correlation appropriately captures the broad pathways of risk sharing across these two clusters of disease traits. When *Q*_*Factor*_ became significant for either the *a*, or the *b* path, we subsequently re-tested the other paths for all individual indicators on the factor to see which of the indicators on the tested factors remained significantly associated with markers and psychiatric disorders or immune-mediated diseases. Only mediation models for indicators with significant *a*, *b*, and *c* paths were then carried forward from the *Q*_*Factor*_ significant factors.

This three-step process thus resulted in mediation models that tested a marker’s mediation of (i) an immune-mediated disease factor to psychiatric factor relationship (from here on denoted as *factor-factor* relationship), (ii) an immune-mediated disease factor to an individual psychiatric disorder relationship or a psychiatric disorder factor to and individual immune-mediated disease relationship (from here on denoted as *factor-trait* relationship), (iii) an individual immune-mediated disease to an individual psychiatric disorder relationship (from here on denoted as *trait-trait* relationship). Mediation models were set up and tested in genomic SEM by specifying “defined” parameters within the *lavaan* syntax for the total and indirect effect of the mediation, which has the advantage of providing standard error on the effects. Total effects were calculated as Total = *a* × *b* + *c’* and the indirect effects were calculated as Indirect = *a* × *b*. An example of this framework as written in *lavaan* syntax is given in S1 Text.

### Multiple regression

While several immune marker pairs exhibited high intercorrelations, our downstream statistical modeling was specifically tailored to their distinct biological relationships. Notably, formal partial-effect testing via multiple regression was applied exclusively to IL-6 and CRP. This decision was driven by biological plausibility rather than statistical redundancy or power constraints. Specifically, IL-6 and CRP function within a consecutive biological cascade, where IL-6 acts upstream to stimulate CRP production and is (unlike CRP) also able to cross the blood-brain barrier [46,47,85]. Testing their partial effects simultaneously allowed us to evaluate the conditional relationships between these biologically intertwined markers and psychiatric outcomes. In contrast, other highly correlated pairs—specifically TNFR1A/TNFR1B and TNF-α/CCL3—represent complementary indicators of parallel immune processes, rather than a sequential cascade. Consequently, these pairs were deemed better suited for joint latent modeling rather than partial-effect testing. Partial effects were estimated for IL-6 and CRP using a multiple regression model in genomic SEM with IL-6 and CRP as two correlated predictors (model specification given in S1 Text). The path diagram for these multiple regressions is given in S2 Fig.

In the case of one marker in a pair consistently exhibiting significant partial effects while the other one did not, we only proceeded to use the significant marker in the mediation models. All multiple regression results were FDR corrected for the number of regressions run for the marker pair.

### Replication

Multiple regression, as well as a, b, and c-path analyses were replicated with independent immune-marker GWAS to assess whether the study’s findings are robust across different datasets. However, only nine out of the 14 included markers were available in an independent dataset and five of the ones that were available were underpowered. This resulted in replications testing the relationship of the genetic liability for Interleukin 1 receptor antagonist (IL-1RA) [86], tumor necrosis factor alpha (TNF-α) [86], C-C motif chemokine 3 (CCL3) [86], and C-reactive protein (CRP) [87] with psychiatric disorders and immune diseases. Additionally, multiple regressions of significant *a* and *b-paths* for CRP were run including IL-6 from the original set of markers along with the independent CRP GWAS. The original IL-6 GWAS was used for replication models with CRP due to the next largest available IL-6 GWAS [86] being underpowered for these analyses. Mediations were then modeled for psychiatric disorder, immune disease, and immune marker combinations that had significant a, b, and c-paths, which was only the association between autoimmune diseases and ADHD as mediated by TNF-α in the replication. We report the results of the replications for the remaining four well-powered markers in the Supplemental Results in S1 Text.

### Mendelian randomization

To support the inclusion of IL-6, disentangle the causal hierarchy of IL-6 and CRP, and isolate their specific direct effects on psychiatric disorders, we performed a series of bidirectional MR and MVMR analyses using the TwoSampleMR package in R [88]. These analyses are described in more detail in the Supplemental Methods in S1 Text.

### Multiple mediators

Multiple psychiatric – immune relationships were significantly mediated by more than one immune marker. This result, alongside the strong genetic intercorrelations of the markers, indicates that some markers likely operate via shared biological pathways. To better understand the total mediation effect of all contributing markers combined, as well as the conditional mediating effects of each marker when accounting for correlated genetic signal, we fit a multiple mediator model to each of these immune-psychiatric relationships in Genomic SEM. For the multiple mediation models, the collective indirect effects were calculated as Indirect_Collective_ = (*a*_*k*_ × *b*_*k*_*) +* (*a*_*j*_ × *b*_*j*_*) +* (*a*_*i*_ × *b*_*i*_*)* with *k*, *j*, and *i* denoting three different markers. The total effects were calculated as Total_Collective_ = *Indirect*_*Collective*_ + *c’*. Model specifications in genomic SEM for the multiple mediation models are given in S1 Text, the path diagram for these multiple mediations is given in S3 Fig.

## Supporting information

S1 Text Supplemental methods. (DOCX)

S1 Table Significant immune disease and psychiatric disorder genetic correlations (c-path). (XLSX)

S2 Table Significant immune mediated disease and immune marker genetic correlations (a-path). (XLSX)

S3 Table Significant psychiatric disorder and immune marker genetic correlations (b-path). (XLSX)

S4 Table Multiple regression results of CRP and IL-6. (XLSX)

S5 Table Model fit for all tested models. (XLSX)

S6 Table Mediation model output of internalizing disorders and mixed pattern diseases as mediated by IL-6. (XLSX)

S7 Table Mediation model output of internalizing disorders and mixed pattern diseases as mediated by IL-1RA. (XLSX)

S8 Table Mediation model output of substance use disorders and mixed pattern diseases as mediated by IL-1RA. (XLSX)

S9 Table Mediation model output of substance use disorders and autoimmune diseases as mediated by TNF-𝛼 and CCL3. (XLSX)

S10 Table Mediation model output of internalizing disorders and mixed pattern diseases as mediated by TNF-𝛼 and CCL3. (XLSX)

S11 Table Mediation model output of substance use disorders and mixed pattern diseases as mediated by TNF-𝛼 and CCL3. (XLSX)

S12 Table Mediation model output of internalizing disorders and mixed pattern diseases as mediated by TNFR1A and TNFR1B. (XLSX)

S13 Table Mediation model output of ADHD and mixed pattern diseases as mediated by IL-6. (XLSX)

S14 Table Mediation model output of internalizing disorders and Crohn’s disease as mediated by IL-6. (XLSX)

S15 Table Mediation model output of ADHD and mixed pattern diseases as mediated by IL-1RA. (XLSX)

S16 Table Mediation model output of ADHD and autoimmune diseases as mediated by TNFR1A + B. (XLSX)

S17 Table Mediation model output of ADHD and mixed pattern diseases as mediated by TNFR1A + B. (XLSX)

S18 Table Sample characteristics of replication immune markers. (XLSX)

S19 Table Significant immune mediated disease and immune marker genetic correlations (a-path) in the replication. (XLSX)

S20 Table Significant psychiatric disorder and immune marker genetic correlations (b-path) in the replication. (XLSX)

S21 Table Multiple regression results of CRP and IL-6 in the replication. (XLSX)

S22 Table Mediation model output of ADHD with autoimmune diseases as mediated by TNF-𝛼 in the replication. (XLSX)

S23 Table Mendelian randomization instruments for IL-6 and CRP. (XLSX)

S24 Table Bidirectional Mendelian randomization and Steiger directionality testing. (XLSX)

S25 Table Multivariable Mendelian randomization sensitivity analyses and results for relevant psychiatric outcomes. (XLSX)

S1 Fig Genetic correlation matrix. Correlation matrix displaying genetic correlations between all included immune and inflammatory markers. Abbreviations for the markers can be found in Table 1.
(TIFF)

S2 Fig Example multiple regression model. Example of a multiple regression model for the effects of CRP and IL6 on the mixed pattern immune disease factor. Single-headed arrows indicate regression relationships as modelled in genomic SEM and importantly do not imply expected or modeled directional relationships.
(TIFF)

S3 Fig Example multiple mediator model. Example of a multiple mediator model for the relationship between an immune disease factor with indicators I1-3 and a psychiatric disorder factor with the indicators P1-3. The markers Marker_1_, Marker_2_, and Marker_j_, serve as mediators and the corresponding *a*, *b-*paths, as well as the overall *c*’-path are tested. With them, the direct effect, the indirect effect for each marker, the indirect_collective_, and total_collective_ effects are calculated according to the above equations. Single-headed arrows indicate regression relationships as modelled in genomic SEM and importantly do not imply expected or modeled directional relationships.
(TIFF)
