# Supplementary material for: Immune markers mediate genetic relationships between immune diseases and psychiatric disorders
Source: PLoS Genet. 2026 Sep 30;22(9):e1012328. doi: 10.1371/journal.pgen.1012328 (PMC13641701; doi:10.1371/journal.pgen.1012328)
Supplement: Supplementary file 1 [file pgen.1012328.s001.docx]

**Supplemental Methods**

**Model Specifications in *lavaan***

In the following we provide the annotated syntax by giving the syntax itself in regular typeset and the annotations in italics. The indicator loadings of two-indicator factors were restricted to be equal and for single indicator factors we constrained the residual variance to 0. The correlations between the factors are automatically modeled in genomic SEM.

***Factor-Factor correlations (c-path)***

Immune-psychiatric factor correlations were tested in genomic SEM by specifying them as following in the *lavaan* syntax:

*Defining the immune and psychiatric factors with their corresponding immune diseases and psychiatric disorders.*

*Immune diseases:*

Immune =~ SLE + T1D + RA + JIA + AD + MG

Mixed =~ e * PSO + e * PSA

Inflamm =~ f * CD + f * UC

Celiac =~ CEL

CEL ~~ 0 * CEL

*Psychiatric disorders:*

Comp =~ AN + OCD + TS

SB =~ g * SCZ + g * BIP

Neuro =~ PTSD + ADHD + MDD + TS + ASD

Int =~ PTSD + MDD + ANX

SUD =~ ALCH + CUD + OUD

*Factor correlations:*

Immune ~~ Comp

Immune ~~ SB

Immune ~~ Neuro

Immune ~~ Int

Immune ~~ SUD

Mixed ~~ Comp

Mixed ~~ SB

Mixed ~~ Neuro

Mixed ~~ Int

Mixed ~~ SUD

Inflamm ~~ Comp

Inflamm ~~ SB

Inflamm ~~ Neuro

Inflamm ~~ Int

Inflamm ~~ SUD

Celiac ~~ Comp

Celiac ~~ SB

Celiac ~~ Neuro

Celiac ~~ Int

Celiac ~~ SUD

***Factor-Marker correlations (a-path and b-path)***

To test for significance in the *a-path* immune disease factors were specified to correlate with all immune markers:

*Defining the immune disease factors with their corresponding immune diseases.*

Immune =~SLE + T1D + RA + JIA + AD + MG

Mixed =~ e * PSO + e * PSA

Inflamm =~ f * CD + f * UC

Celiac =~ CEL

CEL ~~ 0 * CEL

*Defining the immune marker as a single indicator factor.*

Immune Marker Factor =~ Immune Marker

Immune Marker ~~ 0 * Immune Marker

*Factor correlations:*

Immune ~~ Immune Marker Factor

Mixed ~~ Immune Marker Factor

Inflamm ~~ Immune Marker Factor

Celiac ~~ Immune Marker Factor

To test for significance in the *b-path* psychiatric disorders factors were specified to correlate with all immune markers:

*Defining the psychiatric disorders factors with their corresponding psychiatric disorders.*

Comp =~ AN + OCD + TS

SB =~ g * SCZ + g * BIP

Neuro =~ PTSD + ADHD + MDD + TS + ASD

Int =~ PTSD + MDD + ANX

SUD =~ ALCH + CUD + OUD

*Defining the immune marker as a single indicator factor.*

Immune Marker Factor =~ Immune Marker

Immune Marker ~~ 0 * Immune Marker

*Factor correlations:*

Immune Marker Factor ~~ Comp

Immune Marker Factor ~~ SB

Immune Marker Factor ~~ Neuro

Immune Marker Factor ~~ Int

Immune Marker Factor ~~ SUD

***Mediation models***

Mediation models were set up and tested in genomic SEM by specifying “defined” parameters within the *lavaan* syntax for the total and indirect effect of the mediation:

*Defining the immune and psychiatric factors with their corresponding immune diseases and psychiatric disorders:*

Immune Factor =~ Immune Disease 1 + Immune Disease 2 + Immune Disease 3

Psychiatric Factor =~ Psychiatric Disorder 1 + Psychiatric Disorder 2 + Psychiatric Disorder 3

*Defining the immune marker factor with its immune marker and setting the residual variance to 0, since this is a single-indicator factor:*

Immune Marker Factor =~ Immune Marker

Immune Marker ~~ 0 * Immune Marker

*Defining the paths in the mediation model with a, b, and c denoting the a-path, b-path, and c-path respectively:*

Immune Marker Factor ~ a * Immune Factor

Psychiatric Factor ~ b * Immune Marker Factor

Psychiatric Factor ~c* Immune Factor

*Naming the* *defined parameters to get explicit estimates and standard errors for the estimates:*

Ind := a * b

Total := a * b + c

***Multiple regression models***

Multiple regression models were specified within lavaan with a psychiatric disorder as an outcome and IL-6 and CRP as two correlated predictors:

*Defining the psychiatric disorders factor with its corresponding psychiatric disorders.*

Psychiatric Factor =~ Psychiatric Disorder 1 + Psychiatric Disorder 2 + Psychiatric Disorder 3

*Modelling the multiple regression with IL6 and CRP as two correlated predictors.*

Psychiatric Factor ~ IL6 + CRP

*The correlation between IL6 and CRP are automatically modeled in genomicSEM, but are explicitly stated here.*

IL6 ~~ CRP

***Multiple mediation models***

Multiple mediation models with the psychiatric disorder – immune disease relationships mediated by multiple immune markers were specified within lavaan as follows:

*Defining the immune and psychiatric factors with their corresponding immune diseases and psychiatric disorders:*

Immune Factor =~ Immune Disease 1 + Immune Disease 2 + Immune Disease 3

Psychiatric Factor =~ Psychiatric Disorder 1 + Psychiatric Disorder 2 + Psychiatric Disorder 3

*Defining the immune marker factors with their corresponding immune markers:*

Immune Marker Factor 1 =~ Immune Marker 1

Immune Marker 1 ~~ 0 * Immune Marker 1

Immune Marker Factor 2 =~ Immune Marker 2

Immune Marker 2 ~~ 0 * Immune Marker 2

Immune Marker Factor 3 =~ Immune Marker 3

Immune Marker 3 ~~ 0 * Immune Marker 3

*Defining the paths in the mediation model.*

*a-paths:*

Immune Marker Factor 1 ~ a1 * Immune Factor

Immune Marker Factor 2 ~ a2 * Immune Factor

Immune Marker Factor 3 ~ a3 * Immune Factor

*b-paths:*

Psychiatric Factor ~ b1 * Immune Marker Factor 1 + b2 * Immune Marker Factor 2 + b3 * Immune Marker Factor 3

*c-path:*

Psychiatric Factor ~ c_prime * Immune Factor

*Covariances between mediators to capture their genetic overlap*

Immune Marker Factor 1 ~~ Immune Marker Factor 2

Immune Marker Factor 1 ~~ Immune Marker Factor 3

Immune Marker Factor 2 ~~ Immune Marker Factor 3

*Defining indirect and total effects to calculate the specific indirect effects and the total indirect effect based on the estimated parameters.*

*Specific Indirect Effects:*

indirect_Marker1_specific := a1 * b1

indirect_Marker2_specific := a2 * b2

indirect_Marker3_specific := a3 * b3

*Total Indirect Effect:*

total_indirect := (a1 * b1) + (a2 * b2) + (a3 * b3)

*Total Effect (direct effect + total indirect effect)*

total_effect := c_prime + total_indirect

***Replication***

To test whether our findings replicate in an independent dataset, we identified nine of our 14 immune markers that had available GWAS summary statistics in an independent dataset (i.e. outside of the UKBiobank and CHARGE for CRP, and outside the UKBiobank for all other markers). Of these nine available markers, only Interleukin 1 receptor antagonist (IL-1RA)^1^, tumor necrosis factor alpha (TNF-α)^1^, C-C motif chemokine 3 (CCL3)^1^, and C-reactive protein (CRP)^2^ were well-powered enough with a SNP-based heritability ($h_{SNP}^{2}$) *Z*-statistic > 4 (sample characteristics given in **Supplemental Table S18**). The a, b-path analyses, the corresponding mediation analysis, and the multiple regressions were replicated with these independent immune-marker GWAS. Because Interleukin 6 (IL-6)^1^ was underpowered in the replication sample ($h_{SNP}^{2}$ Z-statistic = 3.2), IL-6 from the original analyses was included in the multiple regression replication analyses. Results for the replication of the a, b-path analyses, the corresponding mediation analysis, and the multiple regressions can be found in **Supplemental Tables S19 – S22**) and are described in the **Supplemental Results**.

***Mendelian Randomization***

To disentangle the causal hierarchy of IL-6 and CRP, and isolate their specific direct effects on psychiatric vulnerabilities, we performed a series of bidirectional and Multivariable Mendelian randomization (MR) analyses using the TwoSampleMR package in R.

***Instrumental Variable Selection and Sensitivity Analyses*.** Genetic instruments for IL-6 and CRP were extracted from large-scale European ancestry GWAS^2,3^. Instrumental variables (IVs) were strictly defined as single nucleotide polymorphisms (SNPs) reaching genome-wide significance (p < 5x10^-8^). To ensure instrument independence, linkage disequilibrium (LD) clumping was performed within the TwoSampleMR package utilizing the 1000 Genomes Project Phase 3 European reference panel, with a stringent threshold of r^2^ < 0.001 over a 10,000 kb window. All instruments were strong as indicated by an F-Statistic > 10^4^ with an average F = 241.85 for IL-6 and F = 126.21 for CRP. A list of all instruments and their F-statistics, indexing the association between the SNP and the exposure, are provided in **Supplemental Table S23**. The SNPs identified as instruments for the immune exposures were also then taken from the psychiatric GWAS for internalizing and substance use factors and ADHD, which were the three outcomes with significant bivariate genetic correlations with both CRP and IL-6. We further ran sensitivity analyses for both the bidirectional MR for CRP and IL-6 and the multivariable MR (MVMR) to test for heterogeneity across instruments via Cochran’s Q and directional horizontal pleiotropy via the MR-Egger intercept test.

***Bidirectional MR and Steiger Directionality Testing*.** Because IL-6 and CRP exist within a complex immunological cascade, we first established their biological hierarchy using bidirectional two-sample MR. It is important to formally distinguish the analytical tools used to estimate the causal effect from those used to verify its direction. We estimated the magnitude and significance of the causal effect of genetically predicted IL-6 on CRP, and vice-versa, utilizing standard MR causal estimators (inverse variance weighted method, MR-Egger, and weighted median models). Conversely, to mathematically verify the primary direction of that established pathway and identify potential reverse causation due to unequal GWAS sample sizes, we applied the Steiger directionality test. Rather than estimating an effect size, the Steiger test acted as a directional validity check; it confirmed the causal direction by testing whether the selected genetic instruments explained significantly more variance in the designated exposure than in the outcome.

However, the heterogeneity and pleiotropy sensitivity analyses for both directions were significant, indicating that the strict exclusion restriction assumptions of univariable MR were violated and MVMR was necessary. By including both, CRP and IL-6 as predictors of the psychiatric outcome, MVMR accounted for these pleiotropic pathways to isolate their direct, independent effects on the psychiatric outcomes. The results of the bi-directional MR for the default MR methods included in the TwoSampleMR package (MR Egger, weighted median, inverse variance weighted, simple mode, and weighted mode), the Steiger directionality test and the results of the sensitivity analyses are provided in **Supplemental Table S24**.

***Multivariable Mendelian Randomization.*** To identify the primary driver between those highly correlated immune markers with respect to psychiatric risk, we performed MVMR. MVMR allows for the estimation of the direct, independent causal effect of an exposure while conditioning on the genetic liability of a highly correlated trait. Prior to conducting MVMR, we first performed univariable MR to estimate the isolated effects of genetically predicted IL-6 and CRP on the psychiatric outcomes. We evaluated the exclusion restriction assumption for each of these univariable models using Cochran’s Q and the MR-Egger intercept test. Because CRP frequently exhibited significant heterogeneity and pleiotropy we proceeded with a joint genetic model. The independent IVs for IL-6 and CRP were pooled, and their joint effects were regressed against the psychiatric outcomes. This joint genetic model served to isolate the specific causal contribution of IL-6 signaling pathways independent of CRP. The results of the sensitivity analyses and the MVMR for all psychiatric disorder outcomes are provided in **Supplemental Table S25**.

**Supplemental Results**

***Replication***

The *a* and *b-path* replication analyses showed that CRP was related to autoimmune diseases, mixed pattern diseases, and Crohn’s disease on the autoinflammatory factor. TNF-α was additionally related to autoimmune diseases. The effect sizes of these associations were very similar to those in the original analyses and can be found in **Supplemental Table S19**. For the *b-path*, CRP, IL-1RA, and TNF-α were significantly associated with psychiatric disorders. CRP was associated with the compulsive, internalizing, and substance use factors, as well as ADHD on the neurodevelopmental factor. TNF-α was significantly associated with ADHD. IL-1RA was associated with the compulsive, internalizing and neurodevelopmental factors, however since this marker did not have any significant associations on the *a-path*, it was not carried forward for additional analyses. The effects for all associations with psychiatric disorders were all in the same direction as the original analyses, however, they were much more similar for CRP than for IL-1RA, and can be seen in **Supplemental Table S20**. While findings for the *a-* and *b-path* did not replicate for CCL3 and did not fully replicate for TNF-α, these markers were much lower powered in the replication than in the original analyses and just barely exceeded the heritability Z-statistic > 4 cutoff.

Multiple regressions of significant *a* and *b-paths* for CRP were run including IL-6 from the original set of markers. Replication multiple regressions closely mirrored findings from the main analyses with IL-6 continuing to have significant partial associations and CRP’s associations becoming insignificant. This finding, in addition to the MR findings led us to drop CRP from all subsequent mediation models. The results of the multiple regressions are given in **Supplemental Table S21**.

One mediation model, namely the association between autoimmune diseases and ADHD as mediated by TNF-α, was tested according to the *a-*, *b-*, and *c-path* requirement. Although this was non-significant, the association between the autoimmune diseases and ADHD was mediated ~31% by TNF-α, with an indirect effect of .04 (SE = .03, p_FDR_ = 1.39e-01), and a direct effect of .09 (SE = .05). Full mediation results are given in **Supplemental Table S22**.

**Supplemental References**

1.	Konieczny MJ, Omarov M, Zhang L, et al. The genomic architecture of circulating cytokine levels points to drug targets for immune-related diseases. *Commun Biol*. 2025;8(1):34. doi:10.1038/s42003-025-07453-w

2.	Koskeridis F, Evangelou E, Said S, et al. Pleiotropic genetic architecture and novel loci for C-reactive protein levels. *Nat Commun*. 2022;13(1):6939. doi:10.1038/s41467-022-34688-6

3.	Eldjarn GH, Ferkingstad E, Lund SH, et al. Large-scale plasma proteomics comparisons through genetics and disease associations. *Nature*. 2023;622(7982):348-358. doi:10.1038/s41586-023-06563-x

4.	Sanderson E, Spiller W, Bowden J. Testing and correcting for weak and pleiotropic instruments in two‐sample multivariable Mendelian randomization. *Statistics in Medicine*. 2021;40(25):5434-5452. doi:10.1002/sim.9133
